# Sanitary Law

**Published:** 1883-06

**Authors:** 


### SANITARY LAW.

We heartily commend to the authorities of Georgia, the following acts of legislation taken from the *New York Sanitarian.* The enactment relating to the practice of medicine in New Jersey is especially commendible; it is a step far in advance of the inefficient, merely registering provision of our own State :

"The law regulating the practice of medicine was amended and made more stringent; the provisions of this law are as follows: No person shall commence or continue to practice medicine or surgery without first filing a copy of his diploma with the clerk of the county in which he shall practice. Any violation of this provision shall be deemed a misdemeanor, and upon conviction the person violating the law shall be fined $2, or imprisoned for a term not to exceed six months, or both, for each prescription made, operation performed, or professional service rendered. Any person who shall have had twenty years' practice in one locality, and who shall file with the county clerk an affidavit setting forth such facts, shall be exempt from the requirements of the law. The county clerk is required to keep a record, open to the public, of all copies of diplomas filed with him, and also to transmit a list of those registering to the State Board of Health. The State Board will, in its annual report, publish a list of all physicians entitled to protection under the law, and thus much good will be accomplished. The old law, quite similar but not so strict as this, was rigidly enforced last year in Essex, Passaic, and other counties, and kept not a few irregular practitioners out of the State.

"The law prohibiting the sale of adulterated foods and drugs was amended and strengthened. The State Board of Health is to appoint analysts and inspectors to aid in its enforcement. An appropriation of $1,000 was made to carry out the provisions of the law. This act contains a very unique provision, one not found in similar laws enacted in other States, or in the English or Canadian adulteration laws; it provides that if upon inspection of food or drugs the same shall be found to be adulterated, the health officer or inspector shall have power to forbid the sale of the article until decision is rendered by the court. This will certainly do much good in certain cases, and we await with interest the result of its enforcement.

"The regular annual discussion and attack on the law to prevent the sale of impoverished milk took place, but resulted rather in a victory for the law. This law has proved of great benefit to the farmers in the northern and eastern part of the State, in that it has kept off the market large quantities of poor milk; it has also done good by reducing the quantity of poor milk sold in our cities.

"The principal objection to the law seems to be that it was rigidly and impartially enforced. There is now in the hands of the Governor an act prohibiting the sale of skimmed milk in cities in the State, but we are not informed as to whether he will sign it or not.

"The law to prevent the sale of dangerous illuminating oils, which did so much good last year, was perfected, and the test fixed at 100° Fahr., flash, instead of having both a flash and fire-test as formerly. The law now is similar to the New York statute."

